# A Novel Dimeric Exoglucanase (GH5_38): Biochemical and Structural Characterisation towards its Application in Alkyl Cellobioside Synthesis

**DOI:** 10.3390/molecules25030746

**Published:** 2020-02-09

**Authors:** Mpho S. Mafa, Heinrich W. Dirr, Samkelo Malgas, Rui W. M. Krause, Konanani Rashamuse, Brett I. Pletschke

**Affiliations:** 1 Protein Structure-Function Research Unit East Campus, Gate House, School of Molecular and Cell Biology University of the Witwatersrand, Johannesburg 2050, South Africa; mpho.mafa@wits.ac.za (M.S.M.); Heinrich.Dirr@wits.ac.za (H.W.D.); 2 Enzyme Science Programme (ESP), Department of Biochemistry and Microbiology, Rhodes University, Grahamstown 6140, South Africa; samkelomalgas@yahoo.com; 3 Department of Chemistry, Rhodes University, Grahamstown 6140, South Africa; R.Krause@ru.ac.za; 4 CSIR Biosciences, Building 18, Brummeria, Pretoria 0001, South Africa; Konanani.Rashamuse@dst.gov.za

**Keywords:** Alkyl cellobiosides, dimeric protein, exoglucanase, GH5_38, transglycosylation

## Abstract

An exoglucanase (Exg-D) from the glycoside hydrolase family 5 subfamily 38 (GH5_38) was heterologously expressed and structurally and biochemically characterised at a molecular level for its application in alkyl glycoside synthesis. The purified Exg-D existed in both dimeric and monomeric forms in solution, which showed highest activity on mixed-linked β-glucan (88.0 and 86.7 U/mg protein, respectively) and lichenin (24.5 and 23.7 U/mg protein, respectively). They displayed a broad optimum pH range from 5.5 to 7 and a temperature optimum from 40 to 60 °C. Kinetic studies demonstrated that Exg-D had a higher affinity towards β-glucan, with a *K*_m_ of 7.9 mg/mL and a *k*_cat_ of 117.2 s^−1^, compared to lichenin which had a *K*_m_ of 21.5 mg/mL and a *k*_cat_ of 70.0 s^−1^. The circular dichroism profile of Exg-D showed that its secondary structure consisted of 11% α-helices, 36% β-strands and 53% coils. Exg-D performed transglycosylation using *p*-nitrophenyl cellobioside as a glycosyl donor and several primary alcohols as acceptors to produce methyl-, ethyl- and propyl-cellobiosides. These products were identified and quantified via thin-layer chromatography (TLC) and liquid chromatography–mass spectrometry (LC-MS). We concluded that Exg-D is a novel and promising oligomeric glycoside hydrolase for the one-step synthesis of alkyl glycosides with more than one monosaccharide unit.

## 1. Introduction

Alkyl glucosides and their derivatives are environmentally friendly compounds that are used as industrially important non-ionic surfactants with high surface activity [1]. Alkyl glucosides are biodegradable and have excellent foaming control, wetting, detergent and emulsifying properties. The structures of alkyl glucosides or alkyl polyglucosides consist of aliphatic alcohols and glucose unit(s) obtained from biomass feedstocks. Industrially, it is difficult to produce pure alkyl mono-glucosides. As a result, complex mixture of alkyl-mono-, di-, tri- and oligo-glucosides are produced. These mixtures are generally called alkyl polyglucosides [2]. The products are characterised by the length of the alkyl chain and the average number of glucose units linked to it, which is referred to as the degree of polymerisation (DP).

The enzymatic synthesis of alkyl glucosides occurs through a process called transglycosylation [3]. In the transglycosylation approach, activated glycosyl donors and an alcohol (as glycosyl acceptor) are used to generate a new glycosidic bond and water acts a competing nucleophile. A number of alkyl glucosides have been produced through enzymatic synthesis. These include, among others, methyl, ethyl, proponyl, butanyl, hexanyl and octanyl-d-glucosides [3,4,5]. Bhatia et al. [6] reported that β-glucosidase is one of the most important enzymes responsible for synthesising alkyl glucosides. Cyclodextrin glycosyltransferase is also known to synthesise alkyl glucosides [7].

Bhatia et al. [6] reported that the glycoside hydrolase family 1 (GH1) β-glucosidase enzyme, with an (α/β)_8_ barrel structure, displayed transglycosylation activity. The authors also suggested that xylanase/cellulase (PDB 2HIS), a family 26 lichenase (PDB 2CIP), β-glucosidase (PDB 1UG6), Cel7A (PDB 4C4C), arabinofuranosidase (PDB 2VRQ) and E-82 xylanase (PDB 2D24) are glycosyl hydrolase enzymes, which also display transglycosylation properties. The glycoside hydrolase family 5 is one of the biggest families in the CAZy database (http://www.cazy.org). Most GH5 enzymes have been reported as hydrolases, which break down different carbohydrates and their conjugates molecules [8,9].

Among the GH5 enzymes, cellulases are a group of well-studied enzymes which generally hydrolyse cellulose. Cellulases consists of endoglucanases (EG, EC 3.2.1.4), exoglucanases/cellobiohydrolases (CBHI, EC 3.2.1.91 and CBHII, EC 3.2.1.176) and β-glucosidases (BGLs EC 3.2.1.21). GH5 exoglucanases hydrolyse cellulose from its chain ends through a retaining mechanism, producing short cellobiosides as products [8]. Under favourable conditions, some exoglucanases can also perform trans-glycosylation reactions [8]. To date, there have been known studies demonstrating transglycosylation reactions with respect to alkyl glucosides with the exoglucanases from GH5 enzymes (http://www.cazy.org/GH5_38.html; [9]).

In the current study, we present a novel oligomeric enzyme from GH5_38, Exg-D, exhibiting both hydrolase and trans-glycosylase activities. The dimeric and monomeric oligomers of the enzyme were purified to homogeneity through affinity and size exclusion chromatographic techniques. The pure oligomers were biochemically and structurally characterised. The transglycosylation activity of Exg-D was used to successfully synthesise methyl-, ethyl- and propyl-cellobiosides using *p*-nitrophenyl cellobioside as a glycosyl donor and several primary alcohols as acceptors.

## 2. Results 

### 2.1. Exg-D Amino Acid Sequence Analysis, Cloning and Transformation 

The 20 amino acids constituting the transmembrane signalling of Exg-D were excluded from its sequence, and the remaining 360 amino acids were used to design an expression plasmid containing the gene sequence of *Exg-D*. The pET-11a plasmid harbouring the *Exg-D* gene was successfully transformed into *E. coli* T7 cells. The single colonies of the *E. coli* T7 cells were selected from the agar plates and grown for 16 h to generate fresh cells. For expression, 1 mM IPTG was used for the expression of Exg-D. To avoid the incorporation of Exg-D in inclusion bodies, two temperatures were tested, and the soluble enzyme showed excellent expression at 30 °C compared to 20 °C.

### 2.2. Purification and Oligomeric Nature of Exg-D 

**Figure 1:**
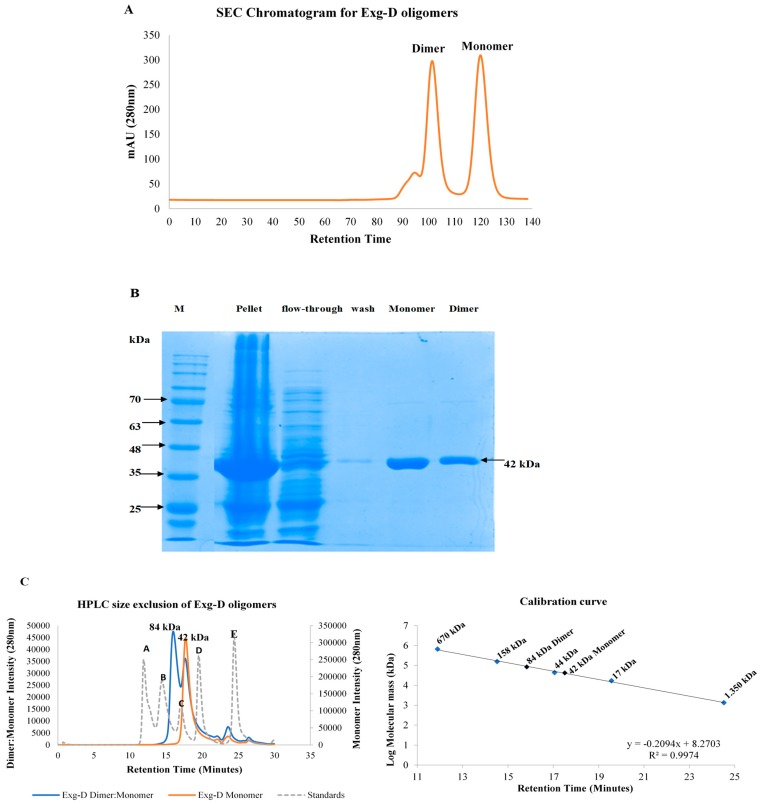
Size exclusion chromatogram of purified dimeric and monomeric forms of Exg-D (**A**). The submolecular sizes of the dimeric and monomeric Exg-D were predicted to be 42 kDa under reducing conditions on SDS-PAGE, as shown in (**B**). Exg-D displayed a homodimer with a molecular mass of about 84 kDa, and the monomer of 42 kDa was confirmed by HPLC size exclusion (**C**). **A, B, C, D** and **E** represent the protein standards with molecular masses of 670,158, 44, 17 and 1.350 kDa, respectively (dotted plot).

Exg-D was purified with a Ni^2+^-charged immobilised metal ion affinity chromatography (IMAC) column. Subsequent to IMAC, Exg-D was further purified to homogeneity using a size exclusion chromatography (SEC) column (HiLoad™ 16/600 Superdex™ 75pg). The SEC results showed that Exg-D exists both as a dimer and a monomer in solution (Figure 1A). These dimer and monomer oligomers were collected separately from SEC. The activities of the dimer and monomer were demonstrated by the colour change of the *p*NPC substrate after addition of the dimeric or monomeric forms of Exg-D. Additionally, the SDS-PAGE profile (under reducing conditions) demonstrated that the dimer and monomer displayed a molecular mass of about 42 kDa for the subunit (Figure 1B). SDS-PAGE results suggested that the dimer is a homodimer consisting of two 42 kDa monomers. HPLC-SE results confirmed that that the dimeric Exg-D oligomer was 84 kDa, while the monomeric oligomer was 42 kDa.

### 2.3. Substrate Specificity

For substrate specificity, both dimeric and monomeric forms of Exg-D were used to hydrolyse the β-1,3-glycosidic linked substrate (pachyman and curdlan), β-1,4-glycosidic linked substrate (carboxymethylcellulose and Avicel PH-101) and mixed-linked β-(1,3)-(1,4)-substrate (lichenin and β-glucan). In lichenin, the β-1,3- or β-1,4-glycosidic bonds are arranged in an alternating fashion forming a 1:2 orientation of mixed linkage. In contrast, β-glucan has one β-1,3-glycosidic bond for every three or four β-1,4-glycosidic bonds, forming a 1:3 or 1:4 orientation of mixed linkage.

The results displayed in Table 1 show that both monomeric and the dimeric forms of Exg-D were highly active on β-glucan, followed by lichenin. Both forms of Exg-D displayed some residual/low specific activity on the β-1,4-glycosidic linked substrate (carboxymethylcellulose and Avicel). However, no enzyme activity was detected on the β-1,3-glycosidic linked substrates (Table 1). These results revealed that the orientation of the β-1,3- and β-1,4-glycosidic bonds in the polysaccharide determined the level of Exg-D activity (e.g., orientations of mixed linkages differs in lichenin and β-glucan). Additionally, the enzyme was not active on a β-1,3-glycosidic linked substrate. These results suggested that presence of the β-1,3-glycosidic bond in the mixed-linkage substrates plays an important role by determining the activity of the Exg-D enzymes.

### 2.4. Biochemical Characterisation and Kinetic Parameters

**Figure 2:**
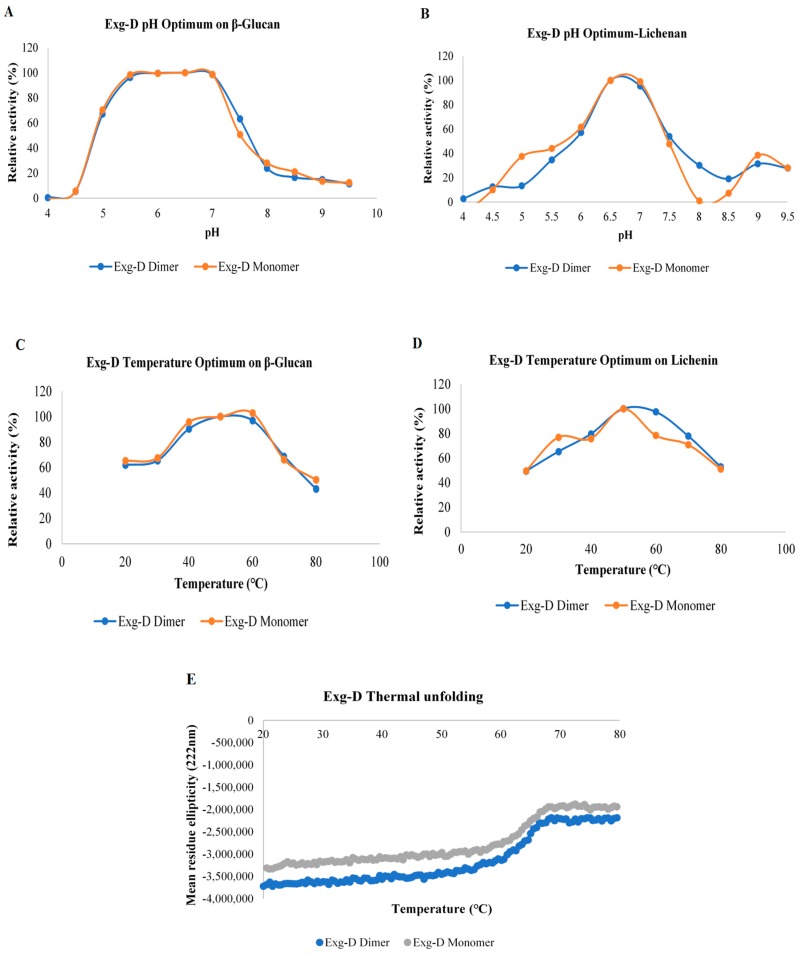
The pH optima of the dimeric and monomeric forms of Exg-D during the hydrolysis of β-glucan (**A**) and lichenin (**B**). The temperature optima of the dimeric and monomeric forms of Exg-D during the hydrolysis of β-glucan (**C**) and lichenin (**D**). The thermal unfolding of the monomeric and dimeric forms of Exg-D (**E**). The experiments were performed in triplicate and error bars represents standard deviations. Experiments were performed in triplicate and the values represent means ± SD.

pH and temperature optima of the dimeric and monomeric forms of the Exg-D were investigated using lichenin and β-glucan. Both oligomeric forms of the Exg-D showed a broad pH and temperature optimum range when they hydrolysed the β-glucan substrate (Figure 2A,C). The pH optima ranged from pH 5.5 to 7 and the temperature optima ranged from 40 to 60 °C. The pH and temperature optima of the monomeric and dimeric Exg-D enzymes were very narrow when the enzyme hydrolysed lichenin compared to β-glucan. The pH optima ranged from pH 6.5 to 7 and the temperature optima ranged from 40 to 50 °C during the hydrolysis of lichenin (Figure 2B,D). These results demonstrated that the differences in the orientation of the β-1,3- and β-1,4-glycosidic linkages in the lichenin and β-glucan substrates affected the performance of both the monomeric and dimeric Exg-D enzymes.

The thermostability of the Exg-D oligomers were tested using circular dichroism (CD). This technique allows for real-time monitoring of protein thermal unfolding, a process which can be used to elucidate the thermostability/thermotolerance of proteins. Thermal unfolding results demonstrated that both the dimeric and the monomeric forms of Exg-D were thermostable from 20 to 60 °C (Figure 2E). The transition state of protein thermal unfolding occurred between 60 to 70 °C. The protein was completely unfolded from 70 to 80 °C, even though there were no signs of protein precipitation (white/milky colour in the cuvette). The protein refolding from 80 to 20 °C was also monitored with CD. The result showed that the protein did not refold back to its native form (data not shown). The refolding results supported the observations that both forms of Exg-D protein were completely unfolded from 70 to 80 °C.

Specific activity, as well as pH and temperature characterisation studies, demonstrated that both the monomeric and dimeric forms of the Exg-D enzyme displayed similar substrate specificity, pH and temperature optima and thermostability properties. However, the monomeric form of Exg-D was the most stable in solution, and the dimers shifted between the dimeric and monomeric form based on the concentration. Thus, the monomeric form of Exg-D was used to determine the kinetic parameter of this enzyme using lichenin and β-glucan as substrates. Due to the high viscosity of the substrates at concentrations higher than 17.5 mg/mL, both the *V*_max_ and the *K*_m_ values of Exg-D were extrapolated from Kaleidagraph (Michaelis–Menten plot) and were validated using Excel-solver software. Exg-D demonstrated a higher affinity and *V*_max_ value towards β-glucan compared to lichenin (Table 2). The *K*_m_ of Exg-D was 7.9 mg/mL on β-glucan compared to 21.5 g/mL when it acted on lichenin. The product turnover number was determined from the slope of the first-order kinetic reaction. Exg-D had a significantly higher product turnover number of about 117.2 s^−1^. These results also supported the observation that the Exg-D enzyme hydrolysed β-glucan more effectively compared to all other tested substrates (Table 2).

### 2.5. Oligosaccharide Product Patterns and Modes of Hydrolysis

**Figure 3:**
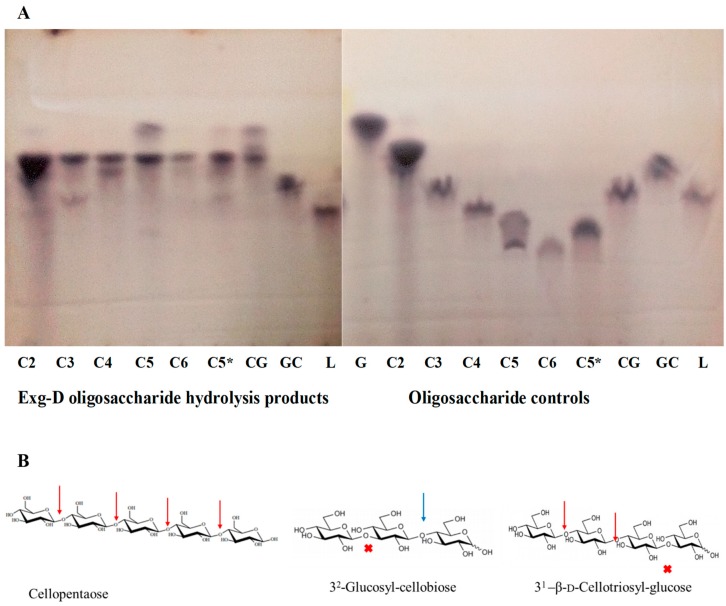
Thin-layer chromatography (TLC) showing the oligosaccharide hydrolysis products of the Exg-D enzyme and the controls (**A**). C2 to C6 represent the cello-oligosaccharides with degrees of polymerisation of 2 to 6, respectively. C3 to C6 were borohydride-reduced, while C2 and C5* were not reduced with borohydride. The CG and GC represent the 3^1^-β-d-cellotriosyl-glucose and 3^2^-glucosyl-cellobiose, while L represents the laminaripetaose. Cellopentaose, 3^2^-glucosyl-cellobiose and 3^1^-β-d-cellotriosyl-glucose were used to indicate Exg-D hydrolysis cleavage sites (β-1,4-glycosidic bonds (red arrows)) and the sites that were not hydrolysed by the enzyme (β-1,3-glycosidic bonds (red cross)) (**B**). The blue arrow shows the potential hydrolysis cleavage site (β-1,4-glycosidic bonds) but the requirement for Exg-D hydrolysis action is two β-1,4-glycosidic bonds, not one.

Exg-D hydrolysed β-1,4-glycosidic bonds in both cellulose substrates and mixed-linkage substrates. To elucidate which of the bonds (β-1,3- or β-1,4-glycosidic bonds) were cleaved during the hydrolysis of the mixed-linkage substrates, three oligosaccharides (β-1,4-D-cello-oligosaccharides, β-1,3-D-laminaripentaose and β-(1,3)-(1,4)-D-mixed-linkage oligosaccharides) with different chemical linkages were subjected to the hydrolysis action of Exg-D. Figure 3 shows that Exg-D hydrolysed the β-1,4-D-cello-oligosaccharides with a degree of polymerisation (DP) of 3 to 6, as well as the mixed-linkage oligosaccharides (3^1^-β-D-cellotriosyl-glucose (CG)). It is important to note that the sorbitol released during the hydrolysis of cellotriitol (C3) (which is a cellobiose molecule linked to a reduced glucose (sorbitol)) was not converted to furfural by the 5% sulfuric acid used to make Molisch’s reagent. As a result, it could not be detected on the TLC plate using Molisch’s reagent (Figure 3). The Molisch’s stain for TLC converts normal aldo/keto sugars to furans which condensed with two alpha-naphtol molecules to give off the blue-black coloured spots on the plates.

There was no enzyme activity detected on β-1,3-D-laminaripentaose, indicating that Exg-D only hydrolysed the β-1,4-glycosidic bonds of the substrates. These observations (β-1,4-D-cello-oligosaccharide cleaving activity and no activity on laminaripenaose) helped to explain why the enzyme had shown residual activity on the polymeric cellulose substrates. Secondary observations (3^1^-β-D-cellotriosyl-glucose activity and no activity on 3^2^-glucosyl-cellobiose) demonstrated the importance of the β-1,3- and β-1,4-D-glycosidic bond orientation in the substrate) (Figure 3). Hence, we propose that the requirement of Exg-D to perform hydrolysis is at least one β-1,3-glycosidic bond followed by two or more β-1,4-D-glycosidic bonds in the backbone chain of the mixed-linkage substrates or β-1,4-D-cello-oligosaccharides with a DP between 3 and 6.

### 2.6. Exg-D Secondary Structure Analysis and 3D Modelling

**Figure 4:**
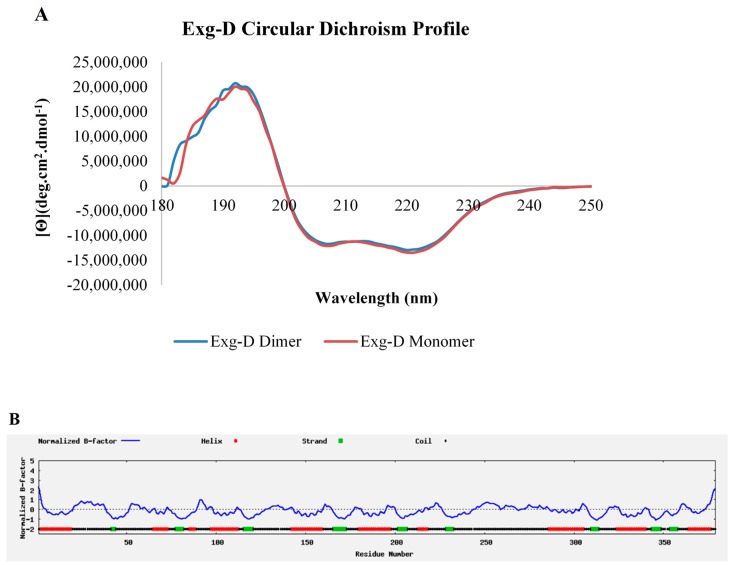
The secondary structure of the dimeric and monomeric forms of Exg-D. The circular dichroism profile shows that both forms of the Exg-D enzyme existed as α-helices and β-strands and coils (**A**). I-TASSER predicted that the Exg-D secondary structure consisted mostly of α-helices, β-strands and coils (**B**). Circular dichroism experiments were performed in triplicate and the values represent the means ± SD.

The secondary structures of the monomeric and dimeric forms of Exg-D were elucidated via CD spectropolarimetry (J-1500, Jasco). The CD spectropolarimetry profiles of both the monomer and dimer demonstrated that secondary structure of Exg-D consisted of both α-helices (represented by two troughs at 222 and 208 nm) and β-strands which absorbed at 192 nm (Figure 4A). The Dichroweb results also predicted that the Exg-D secondary structure consists of 34% α-helices, 14.8% β-strands, 11.7% turns and 39.5% unordered/coils.

Exg-D is classified under GH5_38 (GenBank ID: AMO13174.1), and none of the 27 enzymes in subfamily 38 have a solved 3D structure solved through crystallography or NMR. Thus, I-TASSER online software was used to model the Exg-D secondary, 3D structure and solvent accessibility. The findings from I-TASSER validated the Dichrowed results, predicting that there were about 138 (or 36%) residues that formed eight helices and about 40 (or 10.55) residues that formed eight strands of the enzyme (https://zhanglab.ccmb.med.umich.edu/I-TASSER/output/S505239/). Confidence scores of each amino acid residue that represented helices or strands were mostly 9, which is the highest scoring power, suggesting that these amino acids residues are arranged naturally, as predicted by I-TASSER. Additionally, the coils (about 53% of amino acid residues) had a confidence scoring power of more than 5. The B-factor profiles also confirmed that the predicted helices and strands could be a true representation of the real Exg-D secondary structure because their B-factor profile was mostly below zero (Figure 4B). 

The predicted solvent accessibility results demonstrated that most of the helices and the coils could interact with the solvent, while the stands of Exg-D could not interact with the solvent. All the β-strands of the enzyme were scored a prediction value of 0, meaning that they were buried in the centre of the protein and were mostly covered by α-helices, which had a prediction score ranging from 2 to 5 (https://zhanglab.ccmb.med.umich.edu/I-TASSER/output/S505239/).

**Figure 5:**
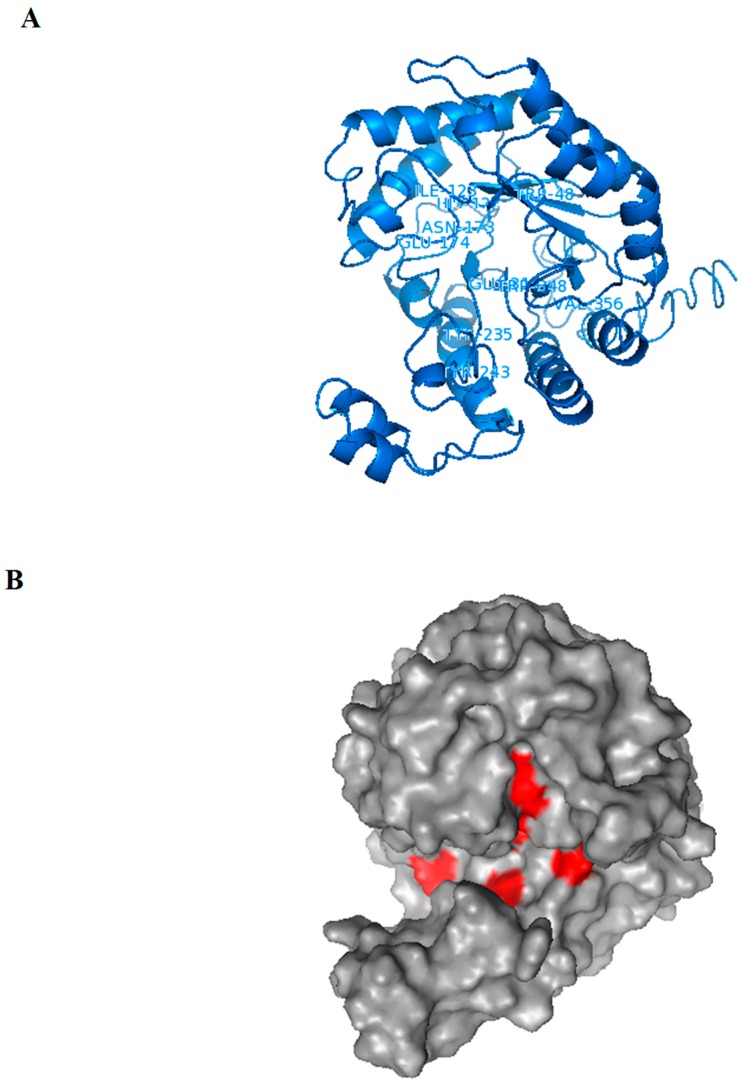
The Exg-D 3D structure model generated using I-TASSER. The structure formed a TIM barrel (β/α)_8_, which is a classical structural for enzymes found in GH family 5 (**A**). The amino acid resides found in the active site (which are predicted to interact with the ligands) are labelled in the cartoon 3D structure (**A**). The surface of the modelled structure shows that Exg-D’s active site forms a tunnel-like-cleft and the positions of amino acids residues which are predicted to interact with the ligands are indicated in red (**B**). The Exg-D modelled structure was visualised and labelled using PyMol.

For the Exg-D 3D modelled structure shown in Figure 5A, the I-TASSER simulations generated a large ensemble of structural conformations called decoys. To select the final models, I-TASSER used a program called SPICKER to cluster all the decoys based on pairwise structure similarity. The program revealed four possible models which corresponded to the four largest structural clusters. Of these four models, the first model had a higher confidence of being the best model with a C-score of −1.20 (Figure 5). Generally, a C-score is typically in the range of [−5, 2], where a C-score of a higher value signifies a model with a higher confidence. The estimated TM-score of the first model was 0.56 (with ±0.15 standard deviation) and an estimated RMSD of 9.4Å (± 4.6Å standard deviation). A TM-score above 0.5 indicates that the modelled structure is of good quality. Additionally, I-TASSER generated fewer than five models, which indicates that the model was of good quality because of the converged simulations. The modelled 3D Exg-D structure consisted of helices, strands and coils as was shown by CD spectropolarimeter results. The modelled structure showed the classical TIM barrel (β/α)_8,_ characteristic of GH family 5 enzymes. The β-strands formed the inner wall, while the α-helices formed the outer wall of the barrel (as shown in Figure 5A)—this structural model coincided with the I-TASSER secondary structure data which scored the β-strands as buried (solvent inaccessible), whereas the α-helices were scored as solvent exposed. The surface analysis of the modelled structure showed that the active site of Exg-D forms a tunnel-like-cleft (Figure 5B). It has been well established that enzymes with this tunnel-like cleft can perform transglycosylation reactions.

### 2.7. Alkyl Cellobioside Synthesis

GH family 5 enzymes (including Exg-D classified as GH5_38) are among the GH enzymes which hydrolyse carbohydrates through a retaining mechanism. Some of the enzymes in this family can also perform transglycosylation reactions when conditions are favourable. To the best of our knowledge, no studies to date have demonstrated that exoglucanases from GH family 5 subfamily 38 can perform transglycosylation reactions. Hence, the current study sought to demonstrate that Exg-D performs transglycosylation, producing alkyl cellobiosides in the process.

#### 2.7.1. Alkyl Cellobioside Synthesis Optimisation 

**Figure 6:**
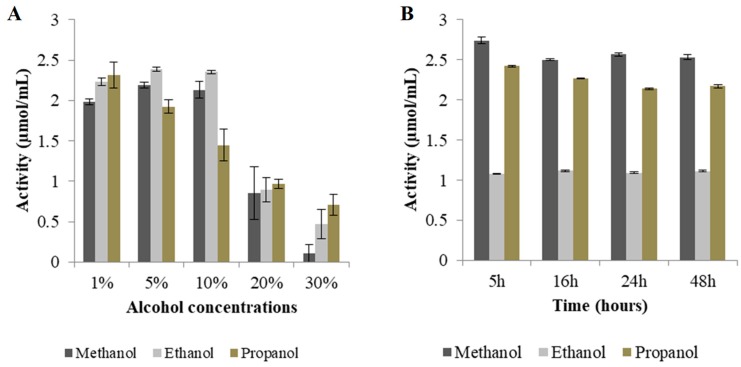

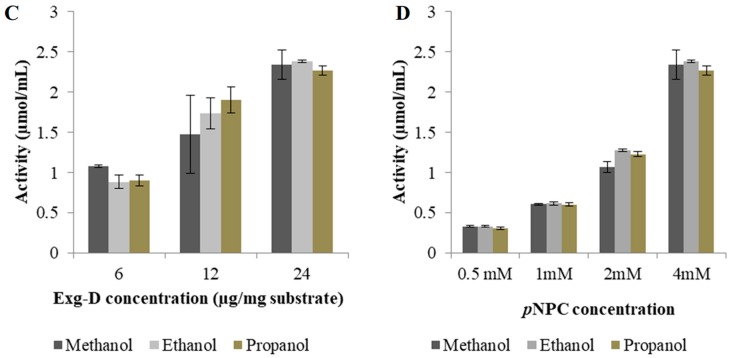
Optimisation of alkyl cellobioside production. The effects of alcohol concentration, time, enzyme, and substrate concentrations are shown in (**A**), (**B**), (**C**) and (**D**), respectively. All experiments were performed in triplicate and the values represent the means ± SD.

The activity of Exg-D was tested using 1.2 mM *p*NPC as substrate. The results showed that the enzyme was active—about 120 µM of *p-*nitrophenol was released after 10 min. After establishing that Exg-D can hydrolyse *p*NPC, conditions were then optimised to allow the enzyme to perform transglycosylation activity. First, the effect of alcohol concentration on Exg-D activity was measured using different concentrations (1 to 30% (*v/v*)) of methanol, ethanol, and propanol, respectively). Low concentrations of alcohols had less of an effect on Exg-D transglycosylation activity, but higher concentrations (>10% (*v/v*)) of alcohol inhibited the enzyme (Figure 6A). Thus, 10% (*v/v*) methanol and ethanol and 5% (*v/v*) propanol were selected as suitable concentrations for further studies. 

Figure 6 shows that there was no significant change in Exg-D activity over time. Based on these results, it was concluded that the reactions should be conducted for less than 5 h. Therefore, all subsequent alkyl cellobioside synthesis reactions were conducted for 2 h. Different enzyme concentrations (6, 12 and 24 µg/mg substrate) were evaluated for optimisation. The results showed that a concentration of about 24 µg/mg substrate exhibited the highest activity of about 2.3 µmol/mL in the presence of 10% (*v/v*) methanol. The addition of 12 µg/mg substrate protein concentration in the presence of 10% (*v/v*) ethanol or 5% (*v/v*) propanol resulted in activities of about 1.73 and 1.90 µmol/mL, respectively (see Figure 6C). High enzyme loadings (24 µg enzyme/mg substrate) only improved the activity by 50% relative to that observed with 6 µg/mg substrate, or 30% relative to that at 12 µg/mg substrate.

The last parameter tested for transglycosylation optimisation was the effect of substrate concentration on Exg-D activity. Figure 6D shows that Exg-D activity increased with increasing *p*NPC substrate concentration. When 4 mM *p*NPC substrate was used, the enzyme displayed higher activity (released more than 2.23 µmol/mL *p*-nitrophenol) compared to 0.5 mM substrate, where the enzyme displayed lower activity, releasing 0.33 µmol/mL of *p*-nitrophenol. Interestingly, Exg-D activity was more than 50% of that at 4 mM *p*NPC in all reactions that contained 2 mM substrate in the presence of methanol, ethanol or propanol. Thus, a 2 mM substrate concentration was selected as the optimal substrate concentration for further studies. 

#### 2.7.2. Identification of Exg-D Transglycosylation Products

The reaction conditions for alkyl cellobioside production were optimised as described above. The products of each optimisation reaction were identified via TLC (Supplementary Figure S1). The TLC profiles supported the optimal alkyl-cellobioside synthesis conditions, which showed that (1) 10% (methanol or ethanol) and 5% propanol, (2) 1 to 6 h reaction time, (3) 6 µg enzyme/mg substrate concentration and (4) 2 to 4 mM substrate concentrations were ideal parameters. TLC demonstrated that Exg-D performed transglycosylation reactions producing methyl-, ethyl- and propyl-cellobiosides, respectively. Additionally, cellobiose was produced as a byproduct in every reaction (see TLC profiles in Supplementary Figure S1).

**Figure 7:**
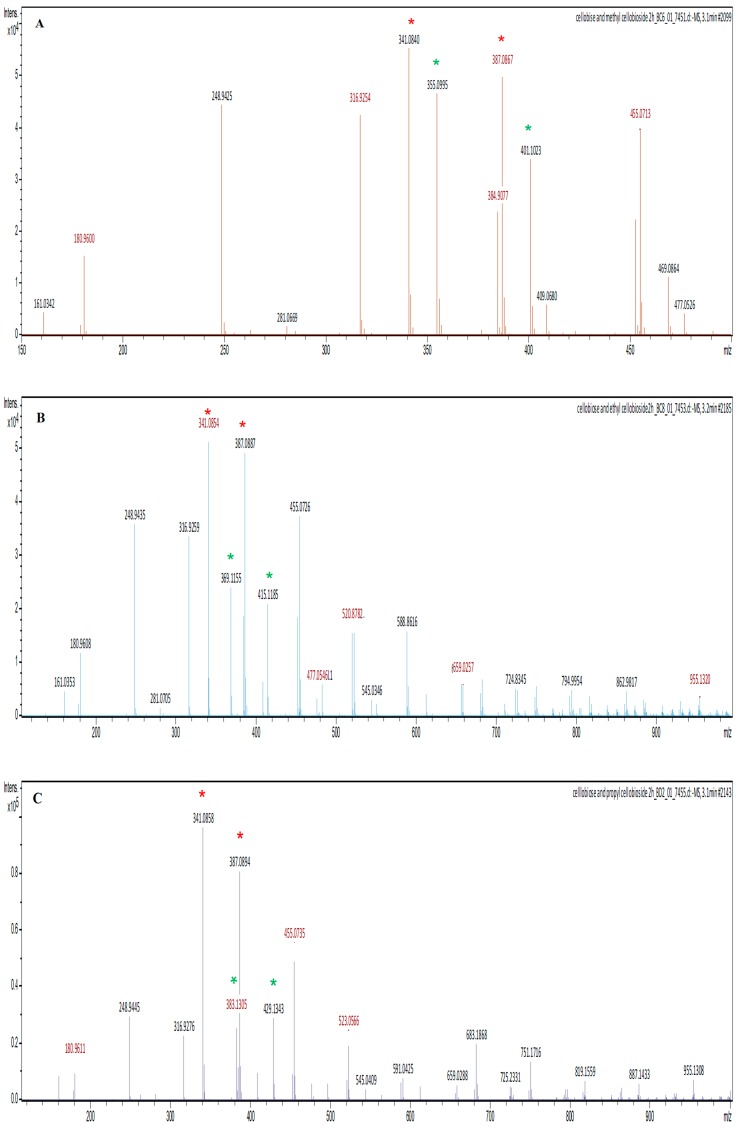
The electrospray ionisation (ESI) profiles of the Exg-D synthesised methyl-, ethyl- and propyl-cellobiosides shown in (**A**), (**B**) and (**C**)**,** respectively. The red stars represent cellobiose and its formic adduct, while the green stars represent alkyl-cellobioside and their formic adducts.

The LC-MS electrospray ionisation (ESI) profiles were used to confirm the identity of the synthesised alkyl cellobiosides and to also quantify their yields relative to cellobiose. The ESI profile (mass to charge ratio (*m/z*)) identified the alkyl cellobiosides and their formic acid adducts, since the mobile phase (50% water (*v/v*) and 50% (*v/v*) acetonitrile) used during LC-MS contained 0.1% formic acid which could be coupled (added) to these analytes. The methyl-cellobioside was identified with an *m/z* of 355.099 since the LC-MS was performed in the negative mode; methyl-cellobioside has a molecular mass of 356.323 Da. The ethyl-cellobioside and propyl-cellobioside components were identified by their *m/z* values of 369.1155 Da and 383.130, respectively (Figure 7). The formic acid adducts of the methyl-, ethyl- and propyl-cellobiosides had *m/z* values of 401.102, 415.1185 and 429.134 Da, respectively. Cellobiose (*m/z* of 341.084) and its formic acid adduct were also identified in three ESI profiles of the synthesized alkyl-cellobiose; cellobiose has a molecular mass of 342.297 Da.

The cellobiose (*m/z* of 341.084) had the highest intensity at about 55,000, while methyl-cellobioside had an intensity of 48,000 (Figure 7). These results suggest that an 87.272% yield of methyl-cellobioside was produced relative to produced cellobiose (assuming that cellobiose at its highest yield equals 100%). Exg-D produced about 55% yield of ethyl-cellobioside and about 30% yield of propyl-cellobioside relative to produced cellobiose. These observations suggest that Exg-D was very effective during transglycosylation reactions of short-chain alcohols, but was not able to donate the sugar molecules to long-chain alcohols such as hexanol (data not shown). Additionally, the yield of the produced alkyl-cellobiosides indicated that Exg-D transglycosylation activity decreased with an increase in the backbone chain of alcohols.

## 3. Discussion

Exg-D is a member of GH family 5 subfamily 38, which includes 27 carbohydrate active enzymes identified to date. Twenty of these enzymes are of bacterial origin (including Exg-D: GenBank ID AMO13174), while seven were unclassified at the time of the current study. Exg-D was sourced from the termite hindgut via metagenomic techniques by Rashamuse et al. [10]. The protein has about 379 amino acid residues, with 21 amino acids constituting the transmembrane signalling of the protein. Tamura et al. [11] demonstrated that the *Bacteroides ovatus* had a GH 16 enzyme that was localised on the membrane and its function was to reduce the length of mixed-linkage-β-(1,3)-(1,4)-glucan to di- or trisaccharides that were transported to the periplasmic region. This shows that the transmembrane signalling sequence of the Exg-D is common among the gut bacterial species and GH enzymes.

The overexpression of Exg-D was optimised using 2× YT liquid Luria-Bertani (LB) medium with 100 µg/mL ampicillin. The results revealed that Exg-D was expressed successfully in T7 *E. coli* cells and the concentration levels were also above 1.8 mg/mL (30 µM). After expression, Exg-D was purified with a Ni^2+^-charged IMAC column as the enzyme was tagged with polyhistidine. To achieve homogeneity of the pure enzyme, Exg-D samples were further purified with a G75 Sephadex gel filtration column. IMAC columns are widely used to purify polyhistidine-tagged proteins, while gel filtration columns are used to purify proteins and also to demonstrate the quaternary oligomeric state (monomeric or dimeric) of proteins in solution [11,12,13]. Interestingly, the gel filtration results demonstrated that Exg-D exists as both a dimer and monomer in solution (Figure 1).

HPLC size exclusion confirmed that the Exg-D exists as a dimer and a monomer, with molecular masses of 84 kDa and 42 kDa, respectively. The dimer and monomer were easily collected into two different fractions because there was a significant difference in the retention times of the two peaks representing these Exg-D oligomers. Further studies probing the nature of the dimer and the monomer revealed that the dimer fraction always consisted of the dimer–monomer combination. This combination was concentration dependent—during high concentrations, the equilibrium shifted to form more of the dimer and vice versa. In contrast, the collected monomeric fraction from G75 gel filtration did not form the dimer–monomer combination (Figure 1C). The GH enzymes generally occur in more than one form; for instance, the bacterial GH 67 α-glucuronidases and the GH 52 β-xylosidase or fungal GH 30_7 exist as dimeric or monomeric oligomers in solution [12,13,14].

The specific activity of Exg-D revealed that both monomeric and dimeric forms of the enzyme were active. The dimer displayed a higher activity of about two activity units (compared to the monomer) on the tested substrates, except for pachyman and curdlan. The active monomer of the Exg-D enzyme was found to be novel, as several studies have generally shown that dimeric oligomers of the GH enzymes are active, while their monomers are not active or display very low activity [13,15,16,17]. The specific activity results were supported by biochemical characterisation and kinetic studies, which showed that the dimeric and monomeric species of Exg-D displayed the same pH and temperature optima. Both Exg-D enzyme oligomers demonstrated a broad pH and temperature optima range on β-glucan. The kinetics studies of the Exg-D also showed that it had the capacity to break down β-glucan (a *K*_m_ of 7.9 mg/mL and a *k*_cat_ of 117.2 s^−1^) compared to lichenin (a *K*_m_ of 21.5 mg/mL and a *k*_cat_ 70.0 s^−1^). The result confirmed the novelty of Exg-D, which has active monomeric and dimeric forms.

Aspeborg et al. [9] demonstrated that enzymes belonging to GH5_38 are not well characterised; out of 27 proteins in this subfamily, only 3 are partially characterised. Thus, the significance of the present study among other things was to biochemically and structurally characterise Exg-D classified under GH5_38 (GenBank ID: AMO13174.1). The GH16 elongating β-transglycosylase from *Paecilomyces thermophila* (PtBgt16A), which also performed hydrolysis of mixed linkages, showed a pH optimum of 5.5 and a temperature optimum of 60 °C [18]. Additionally, a recent study showed the GH5A enzyme from *Talaromyces leycettanus* JCM12802 to have a high specific activity on a mixed-linkage substrate [19]. The enzyme showed a pH optimum of 3 and a temperature optimum of 75 °C. In contrast, Exg-D showed a broad pH optimum from 5.5 to 7 and a temperature optimum from 40 to 60 °C. The broad pH and temperature optima of Exg-D enzyme make it a potential candidate for the hydrolysis of lichenin- or β-glucan-containing biomass.

The oligosaccharide hydrolysis product patterns showed a novel mechanism by which Exg-D enzyme was employed to break down the β-(1,3)-(1,4)-mixed-linkage oligosaccharides. The 3^1^-cellotriosyl-glucose (G4G4G3G) was effectively hydrolysed by the enzyme, while the enzyme showed no activity on the 3^2^-glucosyl-cellobiose (G3G4G). The findings suggest that at least two β-1,4-glycosidic bonds must be joined to a β-1,3-glycosidic bond for hydrolysis to occur. Tamura et al. [11] proposed that GH16 enzymes hydrolyse mixed-linkage oligosaccharides at the β-1,4-glycosidic bond which follows a β-1,3-glycosidic bond; hence, the GH16 enzymes were able to hydrolyse 3^2^-glucosyl-cellobiose (G3G4G). Additionally, the GH16 enzymes were shown to be prolific hydrolysers of laminari-oligosaccharides [11,18]. In contrast, Exg-D showed lost activity on laminaripentaose. This distinction between GH16 enzymes and Exg-D emphasised the novelty of the mechanism employed by Exg-D during the hydrolysis of mixed-linkage oligosaccharides/substrates. In addition, Exg-D was also highly active on cello-oligosaccharides, and it employed the well-studied processive mechanism of exoglucanases [19].

Exoglucanases are known for hydrolysing their substrates in a processive manner, meaning that the enzyme moves along the backbone of cellulose or glucan, releasing cellobiose [9,18,19]. The processivity of the exoglucanases is linked to their tunnel-like-cleft active site, as demonstrated by Qin et al., [18]. Annamalai et al. [20] also argued that CBH I and CBH II from GH7 and GH6 were highly processive due to the topology of the tunnel-like active sites they possess. The current study structurally characterised a termite-hindgut-bacterial-metagenome-derived exoglucanase, Exg-D. The secondary structure of Exg-D consisted of eight α-helices and eight β-strands which were joined by coils. The modelled 3D structure of the Exg-D enzyme resembled the GH5 structure reported by Davies and Sinnott, [8], which is a classical TIM barrel (β/α). The surface topology of Exg-D demonstrated that the catalytic site of the modelled 3D structure of Exg-D was a tunnel-like cleft. Thus, it allowed the enzyme to hydrolyse its substrate in a processive manner, or possibly to perform trans-glycosylation [21,22].

Exg-D performed the transglycosylation activity as hypothesised based on its structural elements. In the present study, Exg-D synthesised methyl-, ethyl- and propyl-cellobiosides. Several other GH enzymes have also been reported to act as trans-glycosylases, for example β-glucosidase, and cellobiose phosphorylase can produce alkyl glucosides [5,21,23,24,25]. To the best of our knowledge, this is the first study demonstrating that GH5_38 exo-glucanases (Exg-D) can perform transglycosylation. Most enzymes hydrolyse polysaccharides or disaccharides to monosaccharides (particularly glucose) first and then use the produced monosaccharides as glycosyl donors, while alcohol is used as an acceptor [26,27]. In contrast, Exg-D hydrolysed *p*NPC, releasing cellobiose and *p-*nitrophenol. Subsequent to the hydrolysis process, the enzyme donated cellobiose to the alcohols. The ESI results showed that the transfer of the cellobiose to methanol resulted in a higher yield of alkyl cellobiosides compared to when longer chain alcohols were used. The enzyme also synthesised appreciable yields of ethyl cellobioside followed by propyl cellobiose. These results suggest that Exg-D can be used only for the synthesis of these short alkyl cellobiosides, as the enzyme lost its trans-glycosylase activity in the presence of higher/longer alcohol (hexanol) acceptors. These results suggest that the transfer of cellobiose to alcohol molecules with a longer chain requires more energy.

## 4. Experimental Section

### 4.1. Protein Sequence Analysis, Cloning and Transformation

The amino acid sequence of the exoglucanase (Exg-D) was recovered from the NCBI GenBank (sequence ID: AMO13174.1), followed by analysis for a possible transmembrane signal peptide sequence using the SignalP 4.1 Server. This showed that the Exg-D sequence contains a transmembrane signal with about 21 amino acids. The Exg-D sequence without the transmembrane signal was sent to GenScript (Piscataway, New Jersey. USA) to cloning the *Exg-D* gene into a pET-11a plasmid. The pET-11a constructs harbouring the *Exg-D* gene were used to transform *Escherichia coli* T7 competent cells. The *E. coli* T7 competent cells were transformed with 50 ng (2 µL) of pET 11a construct containing the *Exg-D* gene insert, mixed gently and incubated on ice for 30 min. Subsequently, cells were heat-shocked for 45 s at 42 °C. The cells were then placed on the ice for 2 min and SOC media (20 g/L Tryptone, 5 g/L Yeast Extract, 4.8 g/L MgSO_4_, 3.603 g/L dextrose, 0.5g/L NaCl and 0.186 g/L KC) was added to the transformed cells and controls. The cells in the SOC medium were allowed to recover by incubation in a shaker incubator (New Braunswick™ Excella^®^, Hamburg, Germany) with a speed of 225 rpm for 1 h at 37 °C. The transformed cells of about 10 µL were streaked onto 2× YT agar plates (16 g/L tryptone, 10 g/L teast extract, 5 g/L NaCl, 15 g/L agar) containing 100 µg/mL ampicillin and incubated at 37 °C overnight (16 h).

### 4.2. Heterologous Overexpression of Exg-D 

The overexpression of Exg-D was optimised and performed by selecting a single colony of transformed *E. coli* cells. For overexpression of Exg-D, the medium (a 2× YT (16 g/L tryptone, 10 g/L yeast extract, 5 g/L NaCl) supplemented with 100 µg/mL ampicillin) was incubated at 37 °C in a shaking incubator until the cells reached mid-log phase (OD_600_ = 0.6). The overexpression of Exg-D was induced by adding 1 mM isopropyl-β-D-1-thiogalactopyranoside (IPTG) into 500 mL of 2× YT medium containing cells, and the cells were incubated at 30 °C in a standing incubator shaking at 150 rpm for 20 h. The cells were harvested by centrifugation (5000× *g*) for 20 min at 4 °C. The cell pellet was resuspended in the lysis buffers (20 mM NaH_2_PO_4_, 0.5 M NaCl, 1 mM CaCl_2_ and 20 mM imidazole, pH 7.0), lysed through pulse sonication (50 Hz) on ice and centrifuged at 5000× *g* for 20 min to separate the Exg-D soluble protein fraction from the cell debris.

### 4.3. Protein Purification of Exg-D 

Purification of Exg-D soluble protein fraction was achieved by immobilised metal ion affinity chromatography (IMAC) charged with nickel ion (Ni^2+^), followed by size exclusion chromatography (SEC). The crude protein obtained from lysed *E. coli* cells was loaded onto the nickel-charged affinity column (GE Healthcare) using an AKTA-Prime fast pressure liquid chromatography (FPLC) purification system. The IMAC column was equilibrated with the 20 mM sodium phosphate equilibration buffer (20 mM of sodium monobasic phosphate, 20 mM sodium dibasic phosphate, 500 mM NaCl and 20 mM imidazole at pH 7). Subsequently, the column was washed with the equilibration buffer until the absorbance values at 280 nm of the effluent reached baseline to ensure that all unbound proteins were washed from the column. The poly-histidine-tagged Exg-D was eluted from the IMAC column using an elution buffer (20 mM of sodium monobasic phosphate, 20 mM sodium dibasic phosphate, 500 mM NaCl and 300 mM Imidazole at pH 7). Exg-D eluted from the IMAC column was purified further using a SEC column (HiLoad™ 16/600 Superdex™ 75pg) connected to an AKTA-Prime system. The SEC column was equilibrated at a flow rate of 0.5 mL/min with 20 mM sodium phosphate buffer containing 500 mM NaCl at pH 7. Exg-D eluted from IMAC column was loaded on the AKTA-Prime system. The two protein peaks showing the highest absorbance values at 280 nm were collected separately and tested for Exg-D activity with *p*-nitrophenol-β-d-cellobioside (a yellow colour change indicated activity). Exg-D was dialysed with dialyses tubes with a 10 kDa molecular weight cut-off in a 20 mM sodium phosphate buffer at pH 7.0 and stored on ice or 4 °C. The level of purity of Exg-D was analysed via 12% SDS-PAGE [28].

### 4.4. Protein Concentration Determination 

The concentration of purified Exg-D was determined using the absorbance values of the monomer or dimer at 280 nm and the extinction coefficient of the protein (74 499 M^−1^ cm^−1^). A Jasco (V-630 UV-VIS) spectrophotometer was used to measure the concentration of the monomeric or dimeric oligomers Exg-D in a glass cuvette. All measurements were performed in triplicate and the purified protein was subsequently determined using the Beer–Lambert law:

*A = εcl* (1)

where *A* is the absorbance value at 280 nm, *ε* is the molar extinction coefficient of Exg-D at 280 nm (M^−1^ cm^−1^), *c* is the concentration of the protein in M and *l* is the path length of the cuvette (1 cm). The molar extinction coefficient of Exg-D was calculated using the extinction coefficients of tryptophan (W), tyrosine (Y) and cysteine (C) residues [29]:

ε = (nW × 5500) + (nY × 1490) + (nC × 125)(2)

= 5500(10) + 1490(13) + 125(4)

= 74 499 M^−1^ cm^−^^1^ (148 998 M^−1^ cm^−1^ for the dimer)

Absorbance at 280 nm of the protein dilution series was determined by fitting a linear regression line to the absorbance values of Exg-D. All readings were corrected for buffer (20 mM sodium phosphate at pH 7.5).

### 4.5. Analysis of the Oligomeric State of Exg-D in Solution

The dimer and monomer oligomers of Exg-D were further investigated via high-performance liquid chromatography (HPLC: Shimadzu, Duisburg, Germany) and AKTA-Prime (FLPC), respectively. The two peaks that were collected separately from the SEC column were analysed with gel filtration HPLC, using a TSK-Gel S3000WXL column (Tosoh, Tokyo, Japan) at 20 °C. The mobile phase was 20 mM sodium phosphate buffer (pH 7.0) containing 500 mM NaCl, using an isocratic flow rate of 0.2 mL/min for 30 min, and the protein was detected at 280 nm. Aliquots of 20 µL of 1 mg/mg to 5 mg/mg Exg-D enzyme were loaded onto the HPLC system to elucidate the oligomeric nature of this protein. 

### 4.6. Specific Activity Determination 

The specific activity of dimer and monomer oligomers of the Exg-D was tested by using β-1,3-glycosidic linked substrates (pachyman and curdlan from Megazyme), β-1,4-glycosidic linked substrates (carboxymethylcellulose and Avicel PH-101) and mixed-linkage β-(1,3)-(1,4)- substrates (lichenin and β-glucan from Megazyme). The reaction was initiated by mixing 1% (*w/v*) of the substrate with Exg-D (10 µg/mg substrate) in 20 mM sodium phosphate buffer (pH 7) at 37 °C for 30 min. Exg-D activity was determined using a modified 3,5-dinitrosalicylic acid (DNS) method [30]. The modified procedure was performed as described previously [31]. Glucose was used as a suitable standard.

Exg-D activity was also tested using *p-*nitrophenol-β-D-cellobioside (*p*NPC) purchased from Sigma. The reaction was initiated by mixing the 4 mM *p*NPC with Exg-D (10 µg/mg substrate) in 20 mM sodium phosphate buffer (pH 7) at 37 °C for 10 min. The para-nitrophenol released by the enzyme action was measured as described by Malgas et al. [32].

### 4.7. Biochemical Properties and Kinetic Studies

To determine the pH optima of the dimeric or monomeric Exg-D, the enzyme with substrate (10 µg/mg substrate) were incubated in 20 mM citric acid–sodium phosphate buffer at pH 4, 4.5, 5, 5.5; 20 mM sodium phosphate at pH 6, 6.5, 7; and 20 mM Tris-HCl buffer at pH 7.5, 8, 8.5, 9 and 9.5, containing 1% (*w/v*) lichenin and β-glucan, respectively. Temperature optima were also determined by dissolving the 1% (*w/v*) lichenin and β-glucan in 20 mM sodium phosphate buffer at temperatures ranging between 20 and 80 °C. The DNS method was used to determine the total reducing sugars released by enzymatic activity.

The thermostabilities of the dimeric and monomeric Exg-D were assessed continuously from 20 to 80 °C with a circular dichroism (CD) spectropolarimeter (Jasco 1500). To detect the thermal unfolding due to increasing temperature (20 and 80 °C), CD spectral measurements were recorded with a spectral bandwidth of 5 nm, a data pitch of 1 nm and a quartz cuvette with a path length of 2 mm. The spectra were recorded as an average of seven scans at a scan speed of 100 nm/min in triplicate. The protein concentration used was 10 μM in 5 mM sodium phosphate at pH 7 to minimise the signal to noise ratio. The spectra were converted from θ (m.deg) to ΘMRE using the following equation:

(3) $$\left[\Theta \right]=\frac{1000}{cnl}$$

where Θ is the mean residual ellipticity (deg.cm^2^.dmol^−1^), θ is the measured ellipticity (mdeg), *c* is the protein concentration (mM), n is the number of residues in the protein and *l* is the path length (cm).

The enzyme kinetics studies of monomeric Exg-D were performed by varying the concentrations (2 to 17.5 mg/mL) of the β-glucan and lichenin dissolved in 20 mM sodium phosphate (pH 7). The *V*_max_, and the *K*_m_ values of Exg-D were measured under steady-state/zero-order conditions and the *k*_cat_ was measured from first-order conditions. The reactions were performed at 37 °C for 30 min, heated at 100 °C to terminate the reaction and the total reducing sugars were quantified using DNS assays. Kaleidagraph and Excel-solver were used to determine the *V*_max_ and *K*_m_ values, while *k*_cat_ values were derived from the slopes of pseudo-first-order kinetics.

### 4.8. Oligosaccharide Hydrolysis Product Patterns

Oligosaccharides were used to study the hydrolysis product patterns of Exg-D and also to establish which of the following bonds: (1) β-1,3-D-glycosidic bonds, (2) β-1,4-D-glycosidic bonds or (3) mixed-linkage β-(1,3)-(1,4)-D-glycosidic bonds were hydrolysed by these enzymes. The reaction was initiated by mixing Exg-D (10 µg/mg substrate) with 5 mg/mL of the oligosaccharides, and the reaction was carried out for 2 h to allow the substrate to be completely converted to products. The reaction was quenched by heating at 100 °C. Laminaripentose was used to study hydrolysis of β-1,3-D-glycosidic bonds by Exg-D. Cellotriitol, cellotetraitol, cellopentaitol, cellopentaose and cellohexaitol were used to study β-1,4-D-glycosidic bond enzymatic activity. The mixed-linked 3^2^-β-D-glucosyl-cellobiose and 3^1^-β-D-cellotriosyl-glucose substrates were used to investigate the order of bonds and the number of β-1,4-D-glycosidic bond required for hydrolysis/cleavage to occur. The cello-oligosaccharides with the suffix “itol” were reduced with borohydride and all the oligosaccharides were purchased from Megazyme. The hydrolysis patterns were detected using thin-layer chromatography (TLC).

### 4.9. Thin-Layer Chromatography

Thin-layer chromatography was used to identify and confirm the oligosaccharide hydrolysis products of Exg-D. The reaction products were analysed using Silica Gel 60G F254 HPTLC plates (Merck, Darmstadt, Germany). Plates were developed twice with n-butanol:acetic acid:water (2:1:1, *v/v*/v). To detect the oligosaccharides, plates were briefly submerged in methanol containing 5% (*v/v*) sulphuric acid and 0.3% (*w/v*) α-naphthol (Molisch’s reagent). Plates were air-dried and heated at 120 °C for 10 min to develop the chromatogram.

### 4.10. Secondary Structure and 3D Modelling of Exg-D

The secondary structures of the dimeric and monomeric Exg-D were determined with a far-UV CD spectropolarimeter. The CD spectral measurements were performed from 180 to 250 nm at 20 °C using a Jasco-1500 spectropolarimeter as described above (Section 2.4). The Exg-D protein concentration used was 5 μM in 5 mM sodium phosphate at pH 7 to minimise the signal to noise ratio. The spectra were converted from θ (m.deg) to ΘMRE was calculated using Equation (3). The CD spectroscopic data were further quantitatively analysed using the Dichroweb server using Contin-LL (Provencher & Glockner Method) to determine the secondary structural content of the Exg-D [33].

Secondary structural characterisation of Exg-D was performed using the I-TASSER server. The full amino acid sequence of Exg-D was submitted to the I-TASSER server (and was assigned ID S505239) for modelling of the secondary and 3D structure of Exg-D according to References [34,35].

### 4.11. Alkyl Cellobioside Synthesis Assays

The alkyl cellobioside synthesis assays (through transglycosylation) were investigated using *p*NPC as a substrate. The standard reaction mixture contained 5% (*v/v*) alcohol (methanol, ethanol or propanol), 4 mM *p*NPC dissolved in a 50 mM sodium citrate buffer (pH 5.5) and 10 µg of Exg-D. The reaction was measured as described in Section 2.6. The optimal conditions for producing the alkyl cellobiosides were investigated by varying: (1) the reaction time from 1 to 6 h, (2) alcohol concentration from 1% to 30% (*v/v*), (3) enzyme concentration from 6 µg/mg to 24 µg/mg substrate and (4) substrate concentration from 0.5 to 4 mM. The alkyl cellobiosides produced during optimisation were measured qualitatively and quantitatively by TLC, LC-MS and by the *p*-nitrophenol released from the reaction, respectively. 

Liquid chromatography–mass spectrometry (LC-MS) was performed in the negative mode to identify and quantify alkyl cellobiosides. LC-MS used mass to charge ratio for identification of the alkyl cellobiosides and electrospray ionisation (ESI) profiles were obtained for quantification. The instrument used was an Ultimate 3000 Thermo-Scientific UHPLC with a Fortis UHPLC C18 column (1.7 μm, 2.1 × 50 mm) and detection was performed by Bruker Compact QqTOF operated in the negative electrospray ionisation mode scanning from 100–1000 daltons. The mobile phase was 50:50 water: acetonitrile with 0.1% (*v/v*) formic acid. The injection volume was 5 μL of ~2.5–20 mM sample solution dissolved in sodium acetate buffer. The masses of the samples were analysed and quantified with Thermo Scientific™ Dionex™ Chromeleon™ 7.2 Chromatography Data System (CDS) software.

## 5. Conclusions

In conclusion, we demonstrated that Exg-D exists as a dimer and monomer in solution. The enzyme was highly active on a mixed-linked substrate (lichenase activity (EC 3.2.1.73)), as well as on mixed-linked oligosaccharides (EC 3.2.1.73) and cello-oligosaccharides (cellulase/glucanase activity (EC 3.2.1.4)). The monomeric and dimeric forms of Exg-D possess broad pH and temperature optima. The secondary structure of Exg-D and its modelled 3D structure demonstrated that the enzyme has an (α/β)_8_ TIM barrel fold with a tunnel-like cleft active site. Finally, Exg-D performed a transglycosylation reaction (transferase activity (EC 2.4.1.29)) and synthesised methyl-, ethyl- and propyl-cellobiosides. Due to its hydrolysis/transglycosylation activities and novel biochemical properties, Exg-D (GH5_38) could have applications in the food or biotechnological industries.

## Figures and Tables

**Table 1:** Specific activities of Exg-D on substrates linked by different glycosidic linkages.

Substrate	Bonds Linking Glucose Units	Exg-D Monomer (U/mg Protein)	Exg-D Dimer (U/mg Protein)
β-Glucan	β-(1,3)-(1,4)-glycosidic linkage	86.7 ± 0.0	88.0 ± 0.0
Lichenin	β-(1,3)-(1,4)-glycosidic linkage	23.7 ± 0.1	24.5 ± 0.1
CMC	β-1,4-glycosidic linkage	2.6 ± 0.0	3.6 ± 0.0
Avicel	β-1,4-glycosidic linkage	1.0 ± 0.0	1.1 ± 0.0
Pachyman	β-1,3-glycosidic linkage	No activity	No activity
Curdlan	β-1,3-glycosidic linkage	No activity	No activity

1 U represents 1 µmol of total reducing sugar released per minute. The experiments were performed in triplicate—values represent mean ± SD.

**Table 2:** The kinetic parameters of the stable monomeric oligomer of the Exg-D enzyme.

Substrates	*V*_max_ (U/mg Protein)	*K*_m_ (mg)	*k*_cat_ (s^−1^)
β-Glucan	4.2 ± 0.3	7.9 ± 1.1	117.2
Lichenin	2.4 ±0.5	21.5 ± 1.0	70.0

The experiments were performed in triplicate; values represent the means ± SD.

## Supplementary Materials

The following are available online, **Figure S1**. Thin-layer chromatography analysis of alkyl cellobiosides produced through Exg-D trans-glycosylation reaction.
