# Supplementary material for: A Novel Dimeric Exoglucanase (GH5_38): Biochemical and Structural Characterisation towards its Application in Alkyl Cellobioside Synthesis
Source: Molecules. 2020 Feb 9;25(3):746. doi: 10.3390/molecules25030746 (PMC7036808; doi:10.3390/molecules25030746)
Supplement: Supplementary file 1 [file molecules-25-00746-s001.pdf]

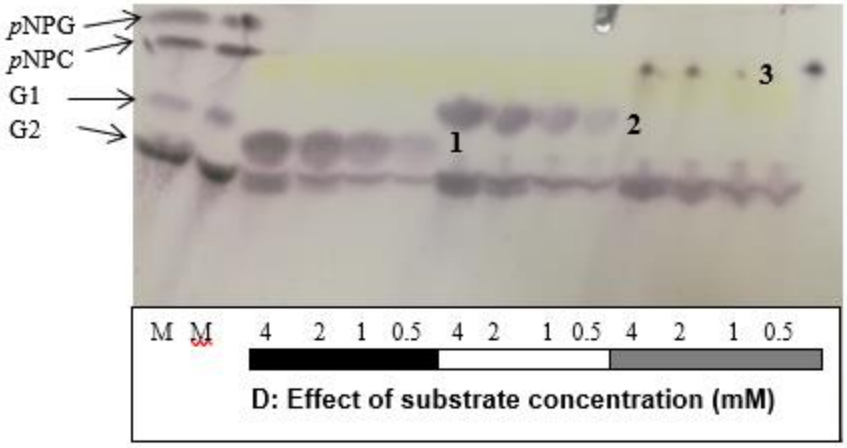

D: Effect of substrate concentration (mM)

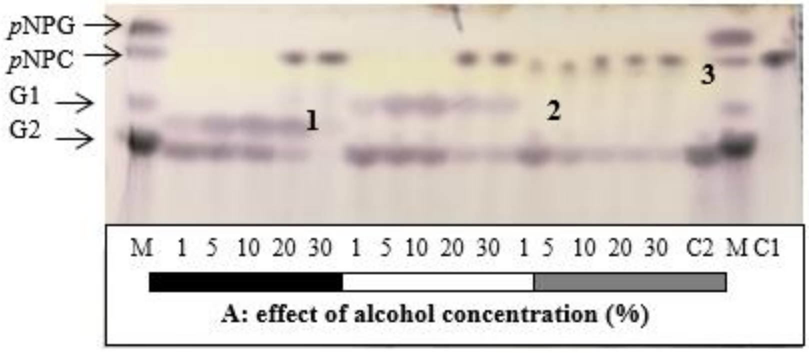

A: effect of alcohol concentration (%)

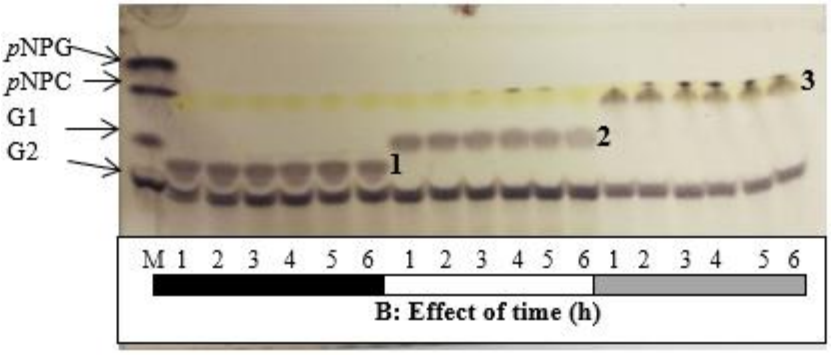

B: Effect of time (h)

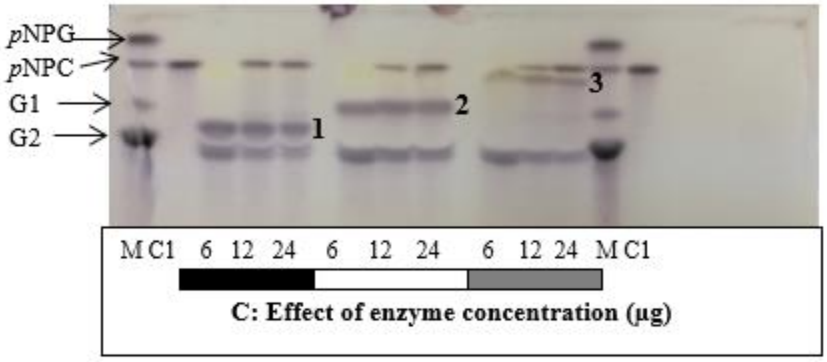

C: Effect of enzyme concentration (µg)

Figure S1. Analysis of alkyl cellobiosides produced through a GH5D trans-glycosylation reaction. The numbers 1, 2, and 3 represent methyl, ethyl, and propyl cellobiosides, respectively. Para-nitrophenyl-glucopyranoside and -cellobioside are represented by *p*NPG and *p*NPC. G1, G2, C1, C2, and M represent glucose, cellobiose, substrate control, reaction control, and standard, respectively. Black, white, and gray bars represent the reactions that contained methanol, ethanol, and propanol as acceptor molecules.
